# Pre-arrest diversion to addiction treatment by law enforcement: protocol for the community-level policing initiative to reduce addiction-related harm, including crime

**DOI:** 10.1186/s40352-021-00134-w

**Published:** 2021-03-10

**Authors:** Aleksandra E. Zgierska, Veronica M. White, Joseph Balles, Cory Nelson, Jason Freedman, Thao H. Nguyen, Sarah C. Johnson

**Affiliations:** 1 Departments of Family and Community Medicine, Public Health Sciences, and Anesthesiology and Perioperative Medicine, Pennsylvania State University College of Medicine, 500 University Dr, Hershey, PA 17033 USA; 2 Department of Industrial and Systems Engineering, University of Wisconsin-Madison, 1513 University Ave, Madison, WI 53706 USA; 3 Safe Communities Madison-Dane County, Inc., 2453 Atwood Ave #209, Madison, WI 53704 USA; 4 City of Madison Police Department, 211 S. Carroll Street, Madison, WI 53710 USA; 5 School of Medicine and Public Health, University of Wisconsin-Madison, 750 Highland Ave, Madison, WI 53705 USA; 6 Public Health Madison & Dane County, 210 MLK Jr Blvd, Room 507, Madison, WI 53709 USA

**Keywords:** Substance use disorder, Addiction, Opioid, Community policing, Crime, Pre-arrest diversion

## Abstract

**Background:**

Despite evidence that treatment reduces addiction-related harms, including crime and overdose, only a minority of addiction-affected individuals receive it. Linking individuals who committed an addiction-related crime to addiction treatment could improve outcomes.

**Methods:**

The aim of this city-wide, pre-arrest diversion program, Madison Addiction Recovery Initiative (MARI) is to reduce crime and improve health (i.e., reduce the overdose deaths) among adults who committed a minor, non-violent, drug use-related offense by offering them a referral to treatment in lieu of arrest and prosecution of criminal charges. This manuscript outlines the protocol and methods for the MARI program development and implementation. MARI requires its participants to engage in the recommended treatment, without reoffending, during the six-month program, after which the initial criminal charges are “voided” by the law enforcement agency. The project, implemented in a mid-size U.S. city, has involved numerous partners, including law enforcement, criminal justice, public health, and academia. It includes training of the police officer workforce and collaboration with clinical partners for treatment need assessment, treatment placement, and peer support. Program evaluation includes formative, process, outcome (participant-level) and exploratory impact (community-level) assessments. For outcome evaluation, we will compare crime (primary outcome), overdose-related offenses, and incarceration-related data 12 months before and 12 months after the index crime between participants who completed (Group 1), started but not completed (Group 2), and were offered but did not start (Group 3) the program, and adults who would have been eligible should MARI existed (Historical Comparison, Group 4). Clinical characteristics will be compared at baseline between Groups 1–2, and pre-post the program within Group 1. Participant baseline data will be assessed as potential covariates. Surveys of police officers and program completers, and community-level indicators of crime and overdose pre- versus post-program will provide additional data on the program impact.

**Discussion:**

By offering addiction treatment in lieu of arrest and prosecution of criminal charges, this pre-arrest diversion program has the potential to disrupt the cycle of crime, reduce the likelihood of future offenses, and promote public health and safety.

## Background

Over the past two decades, the scope and impact of fatal and non-fatal drug overdoses has grown to epidemic proportions in the U.S., with both prescription-based and illicit opioids driving this crisis (Hedegaard, Minino, & Warner, 2020). Between 1999 and 2015, 568,699 Americans died due to overdose; in 2015, 63.1% of 52,404 drug overdose deaths involved an opioid (Scholl, Seth, Kariisa, Wilson, & Baldwin, 2019; Seth, Scholl, Rudd, & Bacon, 2018). This crisis has been paralleled by the increased number of people affected by opioid use disorder (OUD) (Edlund et al., 2014; Saha et al., 2016). Untreated OUD has been linked to increases in criminal justice involvement (Bukten et al., 2012). In 2013, the economic burden related to the opioid crisis was estimated at $78.5 billion, with a third of this amount attributable to negative health consequences of OUD and health care costs, and a quarter related to the public sector cost, including criminal justice (Florence, Luo, Xu, & Zhou, 2016).

Although strong evidence demonstrates that addiction treatment reduces the risk of overdose and other harms, including criminal behaviors, only a minority of people with addiction have accessed treatment, and even a smaller proportion have received live-saving, evidence-based treatment with medications for OUD (MOUD) (Han, Hedden, Lipari, Copello, & Kroutil, 2015; Jones, Campopiano, Baldwin, & McCance-Katz, 2015). Subpar treatment capacity (i.e., insufficient workforce of trained providers) as well as stigma, misperceptions, and lack of knowledge about treatment options and availability among persons with addiction have contributed to the treatment receipt gap (Jones et al., 2015; Robinson & Adinoff, 2018). With a strong relationship between ‘active’ addiction and criminal activity (Bukten et al., 2012), law enforcement are the first-responders who frequently encounter individuals with addiction engaging in drug use-related crime (e.g., theft or drug possession with or without overdose). The ‘traditional’ law enforcement response to addiction-fueled crime has relied on a criminal justice-driven approach (e.g. arrest, prosecution, and/or incarceration).

With longitudinal data indicating substantial reductions in criminal justice involvement among persons engaged in addiction treatment (and lack of reductions outside of such treatment) (Bukten et al., 2012), new approaches to policing have emerged aimed at breaking the cycle of crime and addiction.

In 2015, the Police Assisted Addiction and Recovery Initiative (PAARI) in Gloucester, Massachusetts relied on police officers to assist individuals with a referral to addiction treatment (Schiff et al., 2017). In Seattle, Washington, the Law Enforcement Assisted Diversion (LEAD) program, a community-based, harm-reduction intervention, offered case management, and linkages to supportive services and treatment in lieu of jail and prosecution, leading to reduced recidivism, as compared to the “usual” criminal justice processing (Collins, Lonczak, & Clifasefi, 2017).

The ‘opioid epidemic’ affected all U.S. states, including Wisconsin, where drug overdose-related deaths claimed lives of 878 people in 2015, and 1074 people in 2016 (Hedegaard et al., 2020). The increasing rates of fatal and non-fatal overdoses, and drug use-related crime had stressed the state’s local medical and first-responder infrastructures. To address this escalating crisis, the state’s Attorney General launched in 2015 a nationally recognized awareness and prevention campaign, titled *Dose of Reality* (Wisconsin Department of Justice, 2020). In 2016, the State of Wisconsin Department of Health Services’ *Epidemiological Profile on Alcohol and Other Drug Use* (Wisconsin Department of Health Services, 2016), and the Governor-convened *Task Force on Opioids Abuse* (Task Force on Opioid Abuse, 2016) further underscored the state’s commitment to harm reduction and prevention efforts.

From this momentum, building upon existing research evidence on crime and other harm reduction with addiction treatment, and upon the PAARI and LEAD programs’ early successes, the City of Madison Police Department joined forces with public health, clinical and academic partners, and developed an innovative ‘community policing’ plan, named the *Madison Addiction Recovery Initiative* (MARI). MARI emphasized a collaborative, police-community effort that creates linkages to addiction treatment from police officers as a way for reducing crime and overdoses, and improving lives of individuals with addiction and the local community. In 2016, the MARI proposal was submitted for funding consideration to the U.S. Department of Justice Bureau of Justice Assistance (BJA) Strategies for Policing Innovation (SPI) grant program, which has prioritized development and evaluation of innovative, evidence-based approaches to address ‘chronic’ crime (Bureau of Justice Assistance, 2012). After a competitive peer-review process, a three-year grant was awarded to fund the implementation and evaluation of MARI. This article describes the SPI-approved MARI protocol. The MARI implementation and evaluation findings will be published in subsequent manuscripts.

## Methods

The project design was rooted in the SQUIRE 2.0 standards (Ogrinc et al., 2015), with input from the project’s stakeholder-partners.

### Design

**Fig. 1:**
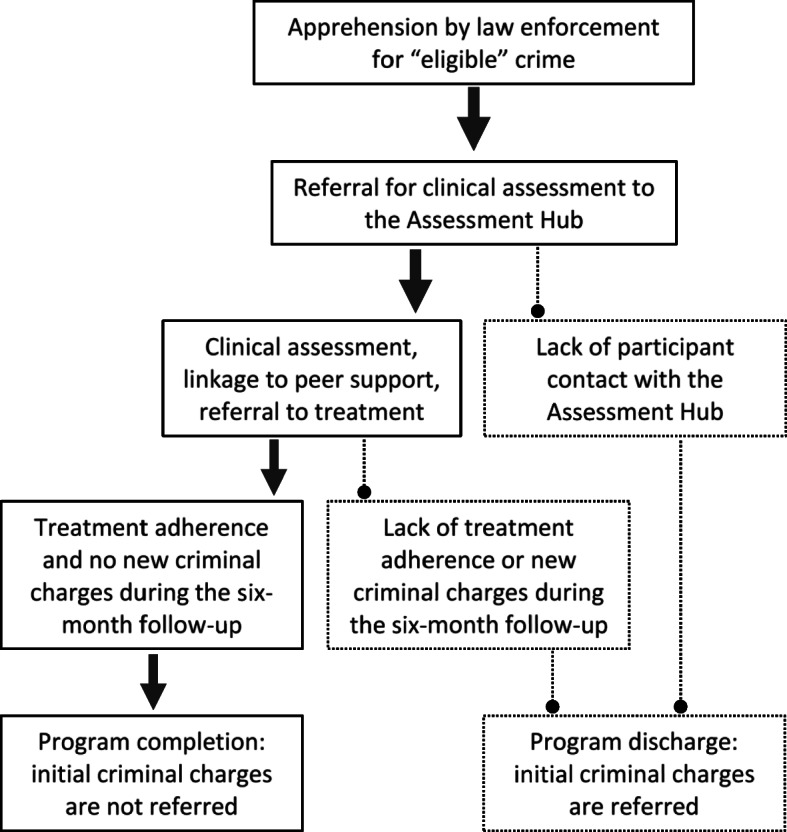
The MARI program design

The primary goal of the MARI program is to reduce crime and crime recidivism of addiction-affected adults, and improve health (i.e., reduce the overdose deaths) by offering treatment to eligible individuals who committed a drug use-related, minor crime in lieu of arrest and prosecution of criminal charges. MARI is a pre-arrest diversion program. Its participants are not initially arrested or charged. Therefore, the official charge is not documented and is held in abeyance through the duration of the MARI program. For program completers, the criminal charges associated with bringing these individuals into MARI are voided (i.e., they do not appear during a criminal background check). Those who do not complete MARI have their formal arrest charges referred to the District or City Attorney’s Offices at the time they leave the program (Fig. 1).The secondary goal is to reduce community-level harm related to untreated addiction, and improve community well-being and safety by connecting eligible individuals to addiction treatment to interrupt the cycle of addiction, overdose, and crime.

### Study timeline

**Fig. 2:**
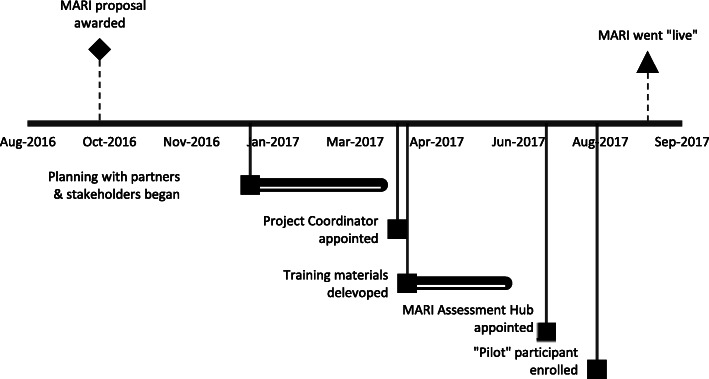
The MARI Development Timeline

The MARI grant award was announced in October 2016 (Fig. 2). The MARI protocol was peer-reviewed by the funding agency reviewers prior to its approval. Following the award announcement, the MARI team started to set up the Operations (Ops) Team and the project implementation plan. The Ops Team is comprised of numerous stakeholder-partners who have been involved in the MARI program from its inception. In early 2017, the Ops Team engaged with additional partners and stakeholders to finalize the procedures for a community-tailored program implementation. The implementation development started by hiring of the MARI Project Coordinator (April 2017); developing training materials, and training of all commissioned law enforcement officers in the MARI protocol and pre-arrest diversion strategy (April–June 2017); and identifying and contracting with the local clinical addiction care provider to serve as the MARI Assessment Hub (July 2017). The MARI Project Coordinator served as a liaison between the MPD, the MARI Assessment Hub, and the evaluation partner. In August 2017, the BJA SPI Program and assigned Subject Matter Experts approved the final project protocol (“Action Plan”) for implementing the MARI project. In August 2017, a “pilot” participant was enrolled to test the project procedures, followed by going “live” with a city-wide participant referral and enrollment on September 1, 2017 (Fig. 2).

We originally planned to complete the participant enrollment period in August 2019. However, to extend recruitment and increase participant sample size, we requested two no-cost project extensions, one in July 2019 (due to the slower than anticipated start of the project rollout), and another in July 2020 (due to the coronavirus pandemic’s impact). The funding agency approved these requests, extending the participant enrollment period through August 2020, and overall funding period through April 2021. The participants enrolled in August 2020 will complete the project’s six-month active follow-up phase in February 2021.

### Setting

The program is supported and approved by numerous city- and county-level organizations, enabling the City of Madison Police Department (MPD) to roll it out across the city, involving all MPD districts. Madison is a medium size city in Wisconsin, USA. The MPD employs 483 commissioned police officers, with approximately 226 assigned to first-responder or patrol duties. Approximately 28% of the MPD officers identify as women, and 86% of the incoming officers completed at least 4 years of college education.

Because addiction is a multi-faceted problem, affecting multiple sectors, collaboration among a wide range of partners has been critical. The MARI Ops Team, responsible for the design, development, and implementation of the MARI program, is comprised of representatives from the MPD (recipient of BJA grant award), city/county public health and human services, a community non-profit injury prevention and advocacy organization, the MARI Assessment Hub, treatment providers, and academic partners.

The program has sought, and been supported by, numerous other local agencies, including the Mayor, Sheriff, District and City Attorney Offices, Emergency Medical Services (EMS), Fire Department, Wisconsin Department of Health Services, and other stakeholders, such as diverse community advocacy groups, treatment providers/programs, and health plans. The perspectives of diverse collaborators and stakeholders have been included throughout the MARI projects. The broader stakeholder-partners and the public have been invited to the quarterly MARI meetings for sharing information, and discussing ways to optimize MARI’s implementation, progress, and sustainability.

### Participant eligibility criteria

Individuals are eligible for MARI if they are adults (18 years old or older), reside in Dane County, and committed an “eligible” non-violent, drug use-related crime (Table 1). The offense-related eligibility criteria were finalized during the program development phase by the MARI Ops Team in collaboration with, and approval from, the County District Attorney’s and the City Attorney’s offices.

**Table 1:** MARI participant eligibility criteria

Inclusion criteria (all need to be met)	Exclusion criteria (none can be met)
• Individual is 18 years old or older; • Individual has ties to Madison or Dane County, WI (i.e., able to participate in the MARI program); • Offense was committed in the jurisdiction of, and handled by MPD, and associated with charges planned to be pressed by MPD; • Offense is non-violent and without a threat of violence; • Offense is drug use-related (crime related to alcohol use only is not eligible); AND • Offense falls into the category of “eligible” ones: o possession of narcotics / drugs or drug paraphernalia (for personal use, not for dealing), prostitution, retail theft, theft from auto without property damage, or burglary / theft from family members who are agreeable to not be pressing charges, or drug overdose	• Presence of valid arrest warrant enforceable in Dane County; • Being on bail for domestic charges or for offenses, which are not MARI eligible; • Being on probation or parole; • Presence of violent felony conviction within the past 3 years; • Being a registered sex offender; • Being deemed as presenting danger to the program staff; • Prior engagement in the MARI program (i.e., completion of the clinical assessment)

The same person could have been referred to MARI more than once. If this person did not engage despite repeated referrals, their initial offense, which led to the first MARI referral, was considered their ‘index crime.’ If this person engaged during the subsequent referral, the offense which led to MARI engagement was defined as their ‘index crime.’ Once they engaged in the program (i.e., completed the clinical assessment), they were not eligible to re-start it; should they re-offend, they could be eligible for drug court or other treatment-oriented diversion programs run by the District Attorney Office, outside of the MARI program.

### Participant timeline

The arresting officers are responsible for conducting an assessment during their investigation to determine if the offending individual meets the MARI eligibility criteria. The MPD officers are trained to identify and assess individuals for MARI eligibility, including whether the committed crime represented a MARI-eligible offense, and was related to addiction. For individuals who are deemed eligible, the arresting officers are directed by the MPD protocols to offer a referral to MARI in lieu of arrest and criminal charges. For individuals who accept the offer and enroll in MARI, criminal charges are held in abeyance (i.e., not pressed or filed). Those who decline MARI are arrested or issued citations by the officer, and their criminal charges are referred to local prosecutorial offices (e.g., District or City Attorney).

After discussing the MARI program, officers review with eligible, interested individuals the MARI referral/consent form, which outlines the program, and provides the program and the Assessment Hub contact information. The referral/consent form is filled out by the arresting officer and signed by the individual. The arresting officer issues and notes on the form all citations, then sends the form to the MPD MARI officer. The MPD MARI officer is then responsible for verifying each referred person’s eligibility. If the individual is deemed ineligible, the MPD MARI officer reaches out to the individual to explain it, rescind MARI participation, file charges, and offer information on existing addiction treatment resources. If a person is incapacitated (e.g., due to an overdose or intoxication), the arresting officer can send the filled out MARI form to the MPD MARI officer who then attempts to contact the eligible person within the next one-to-two business days to discuss the program and offer MARI. The MPD’s trained Sergeants and the MARI officer provide continued oversight and support to officers for MARI-related assessments, decisions, questions and refresher trainings as needed.

Eligible adults who agree to participate in MARI have their charges held in abeyance, and are referred to the MARI Assessment Hub for clinical assessment toward substance use disorders (SUDs) and for peer support (e.g., recovery coaches). The MPD MARI officer securely shares referral information with the MARI Project Coordinator and the Assessment Hub to facilitate the referral. MARI participants are requested to get in touch with the Assessment Hub within three business days to schedule their assessment visit.

The Assessment Hub, a local certified addiction treatment program, was selected through a competitive application process. The Hub provides clinical assessments, facilitates treatment referral and links MARI participants to the Hub’s ‘recovery coaches.’ The Hub’s certified substance abuse counselors complete clinical evaluation to determine the scope and severity of each MARI participant’s SUD-related problems, and optimal level of care, in accordance with the American Society of Addiction Medicine (ASAM) Placement Criteria (Mee-Lee, Shulman, Fishman, Gastfriend, & Miller, 2013). The evaluating counselor then provides each participant with a treatment plan, and helps connect the participant to peer support services, and the recommended treatment services. Treatment plan is tailored to each participant needs, preferences, and health plan coverage. The county Human Services assist with treatment funding for those without health plan coverage. MARI participants sign a ‘release of medical information’ form allowing the Hub counselors to connect and stay in touch with each participant’s treatment provider to track treatment progress during the six-month MARI program. Individuals are deemed as progressing in, and adherent to, treatment (‘yes/no’) based on the treatment provider determination. The MARI officer, Project Coordinator, the Assessment Hub counselors, and a representative from county Human Services are meeting, on average, weekly, to review all “active” MARI participants’ progress.

Participants who adhere to treatment and do not re-offend during the six-month MARI program are considered “Completers” who then receive a Letter of Completion from the Chief of Police and confirmation that their initial charges have been voided by the MPD. Those who do not complete the program (i.e., have not engaged at all, or are discharged due to non-adherence) have their initial charges referred to the City or District Attorney’s Offices at the time of their departure from the MARI program (Fig. 1).

### Evaluation and outcomes

The evaluation plan for the MARI program has focused on answering questions vital to the program itself, the stakeholders, and the long-term program sustainability (Fawcett & Schultz, 2008), such as: A) Formative Evaluation: what factors or processes have been associated with promoting versus hindering the program development and implementation? B) Process Evaluation: what has the program delivered; have participants been satisfied with MARI? C) Outcome Evaluation: how well has the program met its stated objectives, how much and what kind of difference did it make for the participants? D) Impact Evaluation: how much and what kind of difference did it make on the community level? To answer these key questions, several evaluation approaches will assess the MARI project:

A). Formative Evaluation: Information about the program development and implementation will be collected throughout the project to identify the facilitators, barriers, and steps taken to overcome identified barriers to program implementation. This knowledge will enable learning from the project experiences toward optimizing of future implementation on a larger scale, or in different communities. Documentation of community and stakeholder support and engagement, media coverage, and community/system changes associated with progression of the project are indirect measures of the community-level interest in MARI, and can inform sustainability of the MARI approach beyond the grant funding.
B). Process Evaluation: Assessment of participant engagement in the program, the scope of program services, and participant and MPD officer experiences with the program will help better understand factors contributing to program effectiveness, cost, and, ultimately, sustainability and reproducibility.

The MARI Completers, at the time of program exit, complete a survey assessing their satisfaction and experience with the MARI. Police officers completed anonymous surveys in the first year and at the end of the enrollment period of the program. Both participant and officer surveys, developed by the project team specifically for the MARI program, include quantitative questions, with Likert scale responses, and open-ended questions to gather qualitative comments. Descriptive statistics will summarize quantitative data; qualitative analysis methods help analyze the open-ended question comments and identify major qualitative themes.

Program engagement (completion of the clinical assessments, completion of the six-month program among the MARI participants) will help evaluate the willingness to participate and interest among the target population in the MARI program. The Assessment Hub counselor tracks each participant treatment adherence (‘yes/no’) during the program. The MARI MPD Officer monitors each participant’s criminal record for any new offenses; should a new offense occur, the MPD determines if it breeches the MARI program requirements and warrants a participant discharge from the MARI program. Descriptive statistics will describe participant engagement in MARI.

**Fig. 3:**
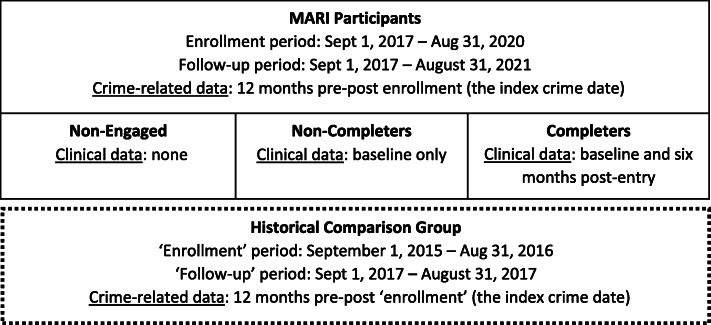
MARI program outcome evaluation design: three subgroups of the MARI participants and a Historical Comparison group

C). Outcome Evaluation (participant level): This evaluation will assess MARI’s impact on individuals. The main goal of the MARI project is to test the hypothesis that facilitating addiction treatment, instead of pressing criminal charges, will lead to reduced crime (primary outcome) and overdose (secondary outcome) among eligible adults who committed minor, eligible crime. The outcome evaluation will be completed by comparing crime-related data of the MARI participants 12 months before and 12 months after their ‘index crime’ date (program enrollment), and by contrasting outcomes between different subgroups of the MARI participants (Non-Engaged: referred but did not engage; Non-Completers: started but did not complete the program; Completers: successfully completed the program), and those in a Historical Comparison group, comprised of adults who would have been eligible for MARI, should this program had existed (Fig. 3). In addition, clinical data, when available, will help evaluate and contrast the baseline clinical characteristics of Non-Completers and Completers, and assess program impact on clinical features of Completers (Fig. 3).

Crime-related data are available for all MARI participants and those in the Historical Comparison group for the 12 months before, and 12 months after the ‘index crime’ (Fig. 3). They are retrieved by the MPD MARI officer from the law enforcement databases, and include data on the number and type of police contacts and arrests, including information on the overdose-related events, and the number of days of incarceration during a given assessment period. Clinical data are available for participants who completed the clinical assessment (Fig. 3), and include ASAM Placement Criteria-based (Mee-Lee et al., 2013), SUD-relevant data collected by the Assessment Hub counselors during the initial clinical assessment (baseline data: both Completers and Non-Completers) and during the final clinical assessment (six-month follow-up data: Completers only). Both crime and clinical datasets include basic demographic information.

MPD MARI officer and the MARI Assessment Hub’s clinical staff securely share the data with the MARI Project Coordinator who links the crime and clinical datasets on a participant level, using the unique identification number, assigned to each participant upon the program entry. The Project Coordinator enters the de-identified data into a password-protected Excel database prior to sharing this de-identified dataset with the academic partners for analyses.

Descriptive statistics will summarize outcome data. A Wilcoxon or paired samples t-test will allow for a comparison of pre-post outcomes within the same group, and a Mann-Whitney or independent samples t-test will compare between-group outcomes for continuous variables. Chi-square or Fisher’s f tests will compare categorical variable-based outcomes. Baseline demographic and, when available, clinical data will be assessed as potential covariates in the analyses.

D). Impact Evaluation (community level): Through improved access to addiction care, the MARI approach, over a longer period of time, has the potential to improve community health and safety, as assessed by the community-level reduced rates of crime, overdose, and overdose-related death, and to reduce related cost. Within the limitations of the MARI project (lack of a comparison community; limited project scope, e.g., due to the restricted MARI eligibility criteria; lack of a sufficient post-MARI follow-up period), the impact of MARI on the community-level indices of safety and health will be of an exploratory nature. Aggregate community-level data will be obtained from the project collaborators who collect such data as a part of their routine duties (e.g., city/county-level data on overdose deaths from Vital Statistics (Wisconsin Department of Health Services, 2020) or naloxone administration for overdose reversal by the first responders, such as MPD or EMS). Pre- and during-MARI community-level data will be contrasted using a similar approach to that described for outcome evaluation.

### Sample size estimates

Based on the MPD’s pre-MARI preliminary data, we estimated that up to 160 adults are apprehended annually for drug use-related minor crimes and could be eligible for MARI.

### Institutional review board

The University of Wisconsin-Madison Institutional Review Board deemed the project “quality improvement,” implementation initiative, and not constituting human subjects research, as defined under 45 CFR 46.102(d).

## Discussion

This article describes the protocol for an innovative, law enforcement-led, city-wide pre-arrest diversion-to-treatment project, which aims to reduce crime and criminal justice involvement, and improve treatment engagement and outcomes among adults with addiction who committed an “eligible” minor, drug-use related crime. Those who enter the program have their charges held in abeyance; after they successfully complete the six-month program by complying with its requirements (staying engaged in addiction treatment, and not re-offending), their initial criminal charges are voided (i.e., not present in any criminal justice databases). The protocol described here offers details on the program structure, so it can be considered by others and improved upon, and serve as a model for implementing similar pre-arrest diversion programs.

MPD has incorporated the MARI diversion protocol city-wide into its standard operating procedures, making it a permanent part of its MPD services, and absorbing the cost to do so. Engaging throughout the project of “key players” and diverse stakeholders who are routinely involved in the prosecution as well as treatment and social services for adult offenders with addiction has been a deliberate step viewed by the project team as essential for the program’s success and long-term sustainability. If effective, the MARI program model could be expanded beyond the city limits, and extended to other individuals who are currently not eligible for MARI but whose untreated addiction might be fueling their criminal activity. For example, MARI focuses on individuals with drug addiction, especially when it involves opioids, which have driven the overdose epidemic. As such, those who committed an alcohol (but not other drug) related crime are not currently eligible for MARI. Similarly, those who are on probation or parole are currently not eligible to enter the MARI program; this may unintentionally negatively impact individuals of color and other groups, which have been disproportionately involved with criminal justice (Williams, Schiraldi, & Bradner, 2019).

Traditional law enforcement, criminal justice-based approaches have been largely ineffective in dealing with criminal offenses committed by individuals who suffer from the disease of addiction (Chandler, Fletcher, & Volkow, 2009). While most law enforcement agencies have basic training in the area of mental health, few law enforcement agencies in the United States have received training specific to addiction, or created and implemented protocols to directly facilitate and connect individuals suffering from mental health disorders and/or addiction to clinical assessment and treatment. Protocols like MARI can help advance a greater understanding and appreciation around the disease of addiction. Although the MARI program’s educational content for police officers has not focused on the ‘science of addiction,’ it has strived to humanize addiction and its treatment, stressed addiction as a chronic brain disease, for which effective, evidence-based treatments are available, and addressed the deleterious impact on treatment engagement of addiction-related stigma. To that extent, the in-service training for all police officers includes both some didactic material, focused on the above themes, as well as testimonies by, and interaction with, individuals in recovery who volunteered for this task. Thousands of officers have been trained in how respond to an overdose incident and *save a life* by administering naloxone (North Carolina Harm Reduction Coalition, 2020). The MARI approach can offer law enforcement officers the opportunity to *change a life* by creating a pre-arrest diversion pathway to assessment and treatment in lieu of arrest and criminal charges. Pre-arrest diversion protocols like MARI can potentially divert countless arrests and referrals for prosecution in the courts for minor offenses, thereby allowing the criminal justice resources to be better focused, and more efficient and timely in regards to individuals who commit higher-risk offenses. In addition, moving away from criminal justice centered approach, which emphasizes punitive charges, toward community policing approach, which is rooted in scientific evidence (i.e., addiction treatment saves lives and improves health), can benefit the communities and their trust in, and relationship with, law enforcement officers.

## Conclusions

The described pre-arrest diversion program, which relies on law enforcement to help link adults who committed minor, drug use-related crimes to addiction treatment, has the potential to improve the health and safety of individuals affected by addiction, and their communities.

## Data Availability

Not applicable.

## Abbreviations

EMS: Emergency Medical Service
LEAD: Law Enforcement Assisted Diversion
MARI: Madison Addiction Recovery initiative
MPD: Madison Police Department
PAARI: Police Assisted Addiction and Recovery Initiative
SUD: Substance Use Disorder
